# Investigating and Analyzing Prognostic Factors and Their Impact on Recurrent Cervical Cancers

**DOI:** 10.7759/cureus.65361

**Published:** 2024-07-25

**Authors:** Ashish Uke, Shweta B Dahake, Anurag Luharia, Monika Luharia, Gaurav V Mishra, Chanrashekhar Mahakalkar

**Affiliations:** 1 Radiation Oncology, Jawaharlal Nehru Medical College, Datta Meghe Institute of Higher Education and Research, Wardha, IND; 2 Medical Physics, Jawaharlal Nehru Medical College, Datta Meghe Institute of Higher Education and Research, Wardha, IND; 3 Medical Physics and Radiology, Jawaharlal Nehru Medical College, Datta Meghe Institute of Higher Education and Research, Wardha, IND; 4 Ayurveda, Jawaharlal Nehru Medical College, Datta Meghe Institute of Higher Education and Research, Wardha, IND; 5 Radiodiagnosis, Jawaharlal Nehru Medical College, Datta Meghe Institute of Higher Education and Research, Wardha, IND; 6 General Surgery, Jawaharlal Nehru Medical College, Datta Meghe Institute of Higher Education and Research, Wardha, IND

**Keywords:** brachytherapy, radiation therapy treatment planning, patient-specific qa, gynecology and obstetrics, external beam radiotherapy (ebrt), uterine cervical cancer

## Abstract

The incidence of cervical cancer in India is significantly high, and the average recurrence age is much less. The standard line of treatment consists of concurrent chemoradiotherapy. If a recurrence occurs, the treatment options or set of interventions are limited and suboptimal. Through this review, we have analyzed and classified the possible prognostic factors for cervical cancer into three broad categories, viz., (a) disease-related factors, (b) patient-related factors, and (c) treatment-related factors. Disease-related factors include tumor histology, tumor size, stage, parametrial involvement (PMI), Prognostic Nutritional Index (PNI), lymphovascular space invasion (LVSI), and nodal status. Patient-related factors include overall treatment time (OTT), nutritional status, hemoglobin level, comorbidities, and age. Treatment-related factors include addition of chemotherapy, techniques of external beam radiotherapy (EBRT), techniques of brachytherapy, and quality assurance for radiation therapy delivery. Out of these, extremely significant prognostic factors were tumor size and stage, nodal status, PMI, nutritional status, and addition of chemotherapy. Impactful factors include younger age, histology, LVSI, associated comorbidities, hemoglobin level, OTT, and patient-specific quality assurance. The factor that is not related or significant is the technique used for EBRT and brachytherapy delivery.

## Introduction and background

Though cervical cancer is a preventable and curable disease, it is a significant cause of death in women in developing countries. In India, as per the GLOBOCAN 2020 statistics, 123,907 new cases were diagnosed, and 77,348 lost their lives [1]. The average age of a patient having cervical cancer recurrence is 40-45 years, and the most important associated etiological factor is human papillomavirus (HPV). According to the International Federation of Gynaecology and Obstetrics (FIGO), the five-year recurrence rate of cervical cancer was 10% for stage IB, 17% for stage IIA, 23% for stage IIB, 42% for stage III, and 74% for stage IVA [2]. The standard treatment modality for cervical cancer consists of surgery, radiotherapy, chemotherapy, or concurrent chemoradiotherapy. The current standard for locally advanced cervical cancer consists of external beam radiotherapy (EBRT) paralleled with cisplatin-based chemotherapy followed by intracavitary/interstitial brachytherapy [3]. However, despite standard treatment, many patients experience local, regional, and distant recurrences. The most common sites for recurrences are the vaginal cuff, followed by the pelvis and other distant sites [4]. In contrast, the most common distant sites are para-aortic lymph nodes, lungs, and supraclavicular lymph nodes [5].

## Review

Method

We have studied more than 90 papers for the review, including original research, review articles, and meta-analyses focusing on various factors associated with cervical cancers and their recurrences. The keywords used for searching through the database were "cervical cancer" AND ("recurrence" OR "radiotherapy" OR "brachytherapy") AND ("treatment" OR "metastasis") AND ("Patient-Specific QA" OR "Planning QA"). One radiation oncologist, two medical physicists, one radiologist, and two physicians individually searched and scrutinized the articles and papers for their relevance to the current topic. The case studies and small studies with insignificant study populations have been excluded from this review. Out of 90, 81 papers were referred for the article and cited.

Discussion 

The search yielded multiple research studies and review articles that have focused on independent prognostic factors, that have only one or two prognostic factors, or that have studied multiple prognostic factors simultaneously in relation to the recurrence of cervical cancer. A deep study and analysis of the available literature showed multiple factors, some extremely significant while others not. We have categorized those into three categories: disease-related, patient-related, and treatment-related. Individual factors with the latest evidence are studied below. 

Disease-related factors

Histology

Histopathology is a keystone in the diagnosis of cervical cancer and has prognostic value. The dominant histological type in cervical cancer is squamous cell carcinoma, and it accounts for 75-80% of all cervical cancers. Adenocarcinoma and adenosquamous carcinoma comprise 10-15%, and the remaining include small cell carcinoma and other unspecified histology. A Surveillance, Epidemiology, and End Results (SEER) population study by Vinh-Hung et al. evaluated 30,989 records of cervical cancer. It concluded that small cell carcinoma and adenocarcinoma are associated with early recurrences and poorer survival, attributed to incidences at young age and inefficient screening policies [6]. A study by Farley et al. included 273 women with cervical cancer, among which 185 have adenocarcinoma histology and 88 have adenosquamous carcinoma histology, from Tripler Army Medical Center, Hawaii, which showed a five-year survival rate of 65% with adenosquamous histology and 83% with adenocarcinoma histology (P<0.002) in the advanced stage but no significant difference in early-stage cervical cancer [7]. In the case of a squamous cell carcinoma of the cervix, the small cell carcinoma antigen level is a good prognostic marker and indicator for recurrences. An original Korean article published in 2019 from Seoul, Republic of Korea, by Cho et al. showed that squamous cell carcinoma is one of the independent prognostic factors for isolated para-aortic lymph node recurrence after extended field radiation therapy (EFRT) [8].

Tumor Size and Stage 

In patients with stage I/II disease, the effect of tumor diameter is not well established because local tumor volume is within the therapeutic range of radiotherapy. Still, when it is more extensive, as in stage III/IV disease, it becomes one of the most important prognostic factors for cervical cancer survival as well as recurrence, as shown in various studies. A study by Kodaira et al. concluded that more than 5 cm tumor size and positive lymph nodes are the independent prognostic factors. They also confirm that a tumor diameter of 6-7 cm seems to be the therapeutic limit for concurrent chemoradiation [9]. A study by Gil-Ibañez et al. on tumor size and oncological outcomes in patients with early cervical cancer treated with once-surgery showed tumor sizes of more than 2 cm had an increased risk of recurrences [10].

Parametrial Involvement (PMI)

PMI is a significant factor in cervical cancer concerning nodal involvement and tumor size. A retrospective study was conducted in Beijing Maternal and Child Healthcare Hospital, China, by Chang et al. with patients of stage IIIC1 with and without PMI. They observed that a patient with PMI has more propensity towards squamous cancer with a higher value of squamous cell carcinoma antigen (SCCA) and higher tumor dimension. Patients with free parametria will likely have adeno histology with lower SCCA levels and smaller tumor dimensions [11]. A Korean study by Lee et al. with stage IB cervical cancer showed that the depth of invasion >1 cm and corpus involvement had a significant relation with PMI and hence poor prognosis. They also suggested an association between tumor size and the involvement of the parametrium [12]. However, Martinelli et al. from the Department of Pathology, IRCCS National Cancer Institute, Milan, Italy, evaluated PMI rate among locally advanced cervical cancer and concluded that PMI had a limited role on cervical cancer recurrences and choice of administration of adjuvant treatment in locally advanced cervical cancer patients undergoing neoadjuvant chemotherapy (NACT) [13]. Winter et al., in their study with a parametrial spread in cervical cancer with negative pelvic lymph nodes, showed that the rate of PMI with a tumor <5 ml, 5-10 ml, and >20 ml was 6.7%, 12.5%, and 33%, respectively. There was no association between the involvement of parametria and the histology of cancer [14]. Kim et al. reported an association between depth of invasion and PMI, showing that patients with stage IB cervical cancer with a depth of invasion of 5 mm are at shallow risk of PMI and fewer recurrences [15]. The other study showing the relationship between PMI and cancer recurrences is mentioned in Table 1.

**Table 1:** Relevant studies showing the relationship of parametrial involvement and cervical cancer recurrences

Sr. no.	Author	Number of patients	P-value
1	Inoue and Okumura [16]	628	<0.001
2	Memarzadeh et al. [17]	93	<0.001
3	Xia et al. [18]	274	<0.001

Lymphovascular Space Invasion (LVSI)

LVSI is a significant predictive factor in the case of cancer prognosis in non-small cell lung cancer, rectal cancer, and head and neck cancer. In the case of cervical cancer, LVSI, along with positive lymph nodes, has a significant negative impact on overall survival (OS) despite the stage at diagnosis. Huang et al. presented a meta-analysis on the prognostic value of LVSI in early-stage cervical cancer, suggesting that LVSI predicts the poor prognostic outcome in stage IA-IIB cervical cancer [19]. The role of LVSI as an independent prognostic factor in early cervical cancer for recurrence and survival in patients with negative lymph nodes was assessed in a study at Radboud University Medical Center, Netherlands, by Pol et al. which included 210 patients with stage IA2 and IB1 cervical cancer. They concluded that satellite LVSI is the most important predictive factor for disease-free survival (DFS) and OS, along with other factors like differentiation grade, tumor size, and conjoined LVSI [20].

Nodal Status

Lymph node metastasis is considered a significant predictor of recurrence and metastasis in cervical cancer. FIGO in 2018 revised cervical cancer staging with the addition of stages IIIC1 and IIIC2 with positive lymph nodes and positive para-aortic lymph nodes, respectively [21]. The factors regarding lymph nodes that predict survival outcomes include (i) nodal size, (ii) location of lymph nodes (pelvic/para-aortic), and (iii) number of positive lymph nodes. Peter's criteria, including positive parametrial invasion, positive margins, and positive pelvic lymph nodes, are indications for the addition of chemotherapy to adjuvant radiotherapy in the case of patients with cervical cancer post-surgical treatment [22]. The study by Uno et al. showed that the five-year OS is 89%, 83%, and 58% in patients with zero positive lymph nodes, one positive lymph node, and two positive lymph nodes [23]. Kato et al. reported a poor prognosis with a five-year OS of 20.2% in patients with three positive lymph nodes [24]. A study by Ditto et al. indicates worsening of OS with increasing positive lymph nodes [25]. Other studies showing a relationship between nodal status and cancer recurrences are shown in Table 2.

**Table 2:** Relevant studies showing the relationship of nodal status and cancer recurrences

Sr. no.	Author	Number of patients	P-value
1	Li et al. [26]	609	<0.001
2	Chen et al. [27]	125	<0.02
3	Teh et al. [28]	120	<0.02
4	Jeong et al. [29]	970	<0.001

Patient-related factors

Overall Treatment Time (OTT)

The OTT for definitive chemoradiotherapy in cervical cancer takes approximately 7-8 weeks, including EBRT and intracavitary brachytherapy. An increase in OTT severely affects OS and, hence, incidents of recurrences. The interval between EBRT and brachytherapy mainly affects DFS. A Taiwanese cohort study by Lin et al., including 2,594 patients with stage I-IVA cervical cancer, found that significant prognostic factors related to poor cancer-specific survival (CSS) and OS included prolonged OTT, advanced age, nonsquamous cell cancer, high-grade histology, increased tumor size, and advanced FIGO stage [30]. A National Cancer Database with 7,355 women with nonmetastatic cervical cancers from 2004 to 2012 which has been reviewed by Hong et al., treated with definitive chemoradiotherapy and brachytherapy, showed that shorter chemoradiation duration is associated with more prolonged survival and should be minimized as much as possible [31]. For further evaluation of reduction in treatment time, a study on accelerated hyperfractionation was conducted by Kavanagh et al. confirming the feasibility and efficacy of concomitant boost accelerated super-fractionated (CBASF) radiotherapy, given as 45 Gy in 25 fractions and an additional 1.6 Gy boost on alternate days for the last three weeks with a cumulative dose of 59.4 Gy. It shows a trend towards improved local control compared to local fractionation, reduced locoregional recurrences, but unacceptable severe late complications [32].

Nutritional Status

Malnutrition and cancer cachexia are associated with a higher rate of post-treatment complications, a low rate of clinical response, and a short survival time. The prognostic nutritional index (PNI), aka Onodera's index, is used as the predominant and principal index in many studies to assess clinical outcomes in cervical cancer [33]. A Chinese systematic review and meta-analysis by Wang et al. included nine promising studies with a total of 2373 patients with early and advanced cervical cancer that showed a stronger relationship between PNI and cervical cancer prognosis and confirmed as independent prognostic factors for recurrent cancers [34]. A small study from Japan by Ida et al. showed PNI as an independent prognostic factor for 12 months, 24 months, and OS (P<0.001) in multivariate analysis [35]. A recent meta-analysis regarding the prognostic and clinicopathological effects of PNI by Niu et al. from Zhejiang University School of Medicine, China, included 2508 cases that showed a low PNI predicted dismal OS, progression-free survival (PFS), and increased propensity of lymph nodal metastasis and concluded as a promising biomarker for the prediction of the prognosis and higher recurrences in clinical practice [36]. Other important and relevant studies are shown in Table 3.

**Table 3:** Relevant studies showing the relationship of the prognostic nutritional index and cancer recurrences

Sr. no.	Author	Number of patients	P-value
1	Haraga et al. [37]	131	<0.002
2	Zhang et al. [38]	235	<0.001
3	Chen et al. [39]	138	<0.05
4	Gangopadhyay [40]	583	<0.0001
5	Chen et al. [41]	138	<0.005

Hemoglobin Level

One of the most common systemic symptoms of cervical cancer is bleeding per vaginum, leading to anemia. Low hemoglobin level is considered a real prognostic factor because of the aggressive biological behavior of the tumor due to relative tumor radioresistance because of tumor hypoxia. Among patients undergoing radiotherapy/chemotherapy pretreatment, hemoglobin levels with optimal oxygen-carrying capacity lead to reoxygenation of hypoperfused tissues, leading to free radical generation and irreversible radiation effects reflecting better tumor control probability (TCP) and better normal tissue control probability (NTCP) [42]. Serkies et al., from the Medical University of Gdańsk, Poland, in 2006, showed a correlation between declining hemoglobin levels during radiotherapy effect and five-year DFS and local control probability (P<0.0001) [43]. Lim et al. from the Department of Radiation Oncology at Perth Radiation Oncology Australia described the outcomes of chemoradiotherapy in cervical cancers, concluding that pretreatment hemoglobin <12 was an adverse prognostic factor for disease recurrence (P<0.03) [44]. Other vital studies showing a significant association between hemoglobin level as prognostic value and tumor recurrences are shown in Table 4.

**Table 4:** Relevant studies showing the relationship of hemoglobin level and cancer recurrence

Sr. no.	Author	Number of patients	P-value
1	Thomas [45]	630	<0.003
2	Grogan et al. [46]	605	<0.0001
3	Winter et al. [47]	494	<0.0001
4	Liu et al. [48]	192	<0.052
5	Shin et al. [49]	805	<0.03

Comorbidities

The Charlson index and Adult Comorbidity Evaluation (ACE)-27 index are used for evaluating comorbidities in cervical cancer. Ferrandina et al. from the Department of Oncology, Catholic University, Campobasso, Italy, evaluated the role of comorbidities in locally advanced cervical cancer and concluded no associated prognostic factor with comorbidities [50]; however, Shin et al. in their study regarding comorbidities in DSF in cancer patients showed that cervical cancer survivors have more comorbidities than the general population and that in turn affect their quality of life (QOL) [51]. The two essential comorbidities associated with cervical cancer outcomes are diabetes and hypertension. Listed below are the associated studies and their relevant impact.

Diabetes: A multivariate analysis on the effects of diabetes and related clinical parameters by Gillani et al., with a total number of 16,946 patients with primary cervical cancer tumors, concluded that type 2 diabetes mellitus (DM) has a significantly higher rate of mortality in both overall mortality (28.3%) as compared to type 1 DM (17.3%) and non-DM patients (12.7%) (P<0.001) [52]. Also, a systematic review and meta-analysis by Chen et al. including 11 studies and 11,091 cervical cancer patients concluded that diabetes was independently associated with poor OS (P<0.001) and poor recurrence-free survival (P<0.001) [53].

Hypertension: A paper studying the correlation of hypertension and hyperglycemia with local invasion of cervical cancers by Shen et al., from Huazhong University of Science and Technology, Wuhan, China, including 246 patients, concluded that hypertension was an independent risk factor for parametrial invasion (OR=6.54) and significantly lower five-year OS rate (P<0.0001) and a significantly lower five-year PFS rate than those without hypertension (P=0.010) [54].

Age

The incidence of cervical cancers is not uniform, having an increasing trend towards advanced age, but it can occur in all age groups. Though many studies showed a relevant association between the older age of the patient and advanced disease, the occurrence of disease at an early age is associated with dismal OS, PFS, DFS, and CSS and more chances of recurrences. In an older study involving 218 patients treated at Loma Linda University between 1972 and 1982, the influence of age on prognosis for cervical carcinoma was evaluated by Spanos et al. The age groups were <35, 35-55, 55-75, and >75 years. As concluded by the authors, age differences did not significantly impact local-regional or distant failures [55]. An actuarial analysis of DFS concluded the same. However, a long-term retrospective cohort study by Li et al. about cervical cancer prognosis and related risk factors, including a total of 4358 patients, showed a lower risk of recurrence in age greater than 60 years (HR 0.53, 95% CI 0.30-0.94) against ages younger than 40 years (P<0.002) [56]. Another study by Wang et al. from China studied a total of 284 patients with recurrent cervical cancer, evaluating and showing an association of tumor appearance, tumor size, and patient age as independent risk factors for early recurrence (P<0.05) [57]. Zhou et al., in a two-way outcome study of 460 patients from Harbin Medical University, Harbin, China, showed primiparous age above 30 years and age at diagnosis below 40 years as poor prognostic factors for OS, PFS, and QOL and early recurrences [58]. Pelkofski et al., in their study at the University of Virginia for cervical cancer patients younger than 35 years of age (n=126), showed that PMI, tumor stage, and tumor histology are strong prognostic factors for PFS and OS [59].

Treatment-related factors

Addition of Chemotherapy

After the publication of five trials in 1999, a recommendation was given by the National Cancer Institute (NCI) stating that "concomitant (cisplatin-based) chemoradiotherapy should be considered instead of radiotherapy alone in women with cervical cancer" [60]. As shown by Green et al., with both platinum and non-platinum chemotherapy, there are an improvement in local control and a highly significant reduction in the rate of distant metastases while evaluating survival and recurrence after concomitant chemotherapy for cervical cancers [61]. A Meta-Analysis Group, Medical Research Council Clinical Trials Unit, London, United Kingdom, showed a 6% improvement in five-year survival with concurrent chemoradiotherapy in treating cervical cancers [62]. Another meta-analysis by Lukka et al. confirms that concurrent cisplatin-based chemotherapy combined with radiotherapy improves OS in locally advanced cervical cancer, large stage IB tumors, and high-risk early-stage disease [63]. Landmark trials stating the role of concurrent chemotherapy in treating cervical cancers, radiotherapy, and its impact on OS, PFS, and recurrence rate are listed in Table 5.

**Table 5:** Relevant studies showing the outcome of addition of chemotherapy to radiotherapy

Sr. no.	Author	Number of patients	P-value
1	Morris et al. [64]	403	<0.004
2	Rose et al. [65]	526	<0.001
3	Keys et al. [66]	183	<0.001
4	Whitney et al. [67]	368	<0.03
5	Peters et al. [68]	268	<0.003

Techniques of EBRT

The technological development in radiotherapy, from bidimensional (2D) techniques like surface anatomy and radiographs to computed tomotherapy and magnetic resonance imaging, 3D image reconstruction, and volumetric-based radiotherapy, improved overall patient TCP with reduced recurrences and NTCP. In a multivariable analysis by Dröge et al. comparing the outcome and toxicity of volumetric modulated arc therapy (VMAT) to conventional 3D conformal radiotherapy (3D CRT), it was found that the survival rate between the treatment groups has no significant difference. On the one hand, whereas VMAT significantly reduced late small bowel toxicity, it was also independently linked with a higher risk of acute urinary toxicity [69]. Kombathula et al. studied the early clinical outcomes in patients of cervical carcinoma treated with VMAT for outcome and toxicity profile. They concluded that VMAT is a preferable treatment modality for cervical cancer. The cervix is considered to have low OAR toxicities; however, longer follow-ups will be needed for long-term disease control and late treatment of toxicities [70]. A meta-analysis by Lin et al. compared the efficacies and toxicities of intensity-modulated radiation therapy (IMRT) with 3D CRT or conventional 2D RT for the definitive treatment of cervical cancer; it was found that there were no significant differences between IMRT and 3D CRT or 2D RT considering the three-year OS and DFS. Still, there was a significant difference between acute gastrointestinal (GI) and genitourinary (GU) toxicities and chronic GU toxicity, where IMRT performed better [71]. Yu et al. from Ningbo Women and Children's Hospital, Ningbo, China, retrospectively analyzed the clinical data of 104 patients where they divided into IMRT vs 3D CRT both followed by intracavitary posterior radiotherapy (brachytherapy); they showed IMRT is equivalent to 3D CRT [72]. Chen et al. studied the clinical outcomes of 68 patients with post-hysterectomy cervical cancer treated with CRT with the IMRT technique. They showed improved locoregional control and lesser toxicities than the conventional box RT technique [73].

Techniques of Brachytherapy

As discussed above, the standard of care for locally advanced cervical cancer includes EBRT combined with brachytherapy. Brachytherapy takes advantage of the inverse square law, which means that the radiation dose is inversely proportional to the square of the distance from the source and rapidly falls off after a certain distance. A higher stage means a locally advanced disease is difficult to control with brachytherapy, leading to more recurrences. A prospective randomized clinical trial from PGI Chandigarh by Patel et al. comparing low dose rate (LDR) vs high dose rate (HDR) intracavitary brachytherapy for the treatment of carcinoma of the uterine cervix including a total of 482 patients concluded that overall local control in the LDR group was 79.7% as compared to 75.8% in the HDR group. In stage I, it was 78% for LDR patients and 78% for HDR patients; for stage II, it was 62% and 64%, respectively; and for stage III, it was 50% and 43%. It showed that HDR intracavitary brachytherapy is equally good as conventional LDR brachytherapy [74].

On the other hand, the final results of Osaka University Hospital for a prospective randomized comparative study of HDR vs LDR therapy by Teshima et al. showed that the five-year cause-specific survival rates of stage I-III patients treated with HDR were 85%, 73%, and 53%, respectively. The corresponding figures for LDR were 93%, 78%, and 47%, respectively. These results confirm equivalent cause-specific survival for LDR and HDR by a higher incidence of complications in the case of HDR [75].

Quality Assurance (QA) for RT

Lack of awareness or maintenance of strict adherence towards quality control (QC) and QA checks for the clinical implementation of any photon radiation (brachytherapy as well as linear accelerator) will lead to the improper delivery and execution of the RT, which in turn will reflect as a non-optimal clinical result. The radiotherapy process's QC is reviewed for potential risks and errors and is guided by various organizations. The medical physicist must perform the QA and QC procedures at a hospital or radiotherapy center. Knöös, Radiation Physics, Skåne University Hospital, Lund, Sweden, and Department of Medical Radiation Physics, Clinical Sciences, Lund University, Lund, Sweden, reviewed the potential risks or pitfalls in modern advanced modalities such as IMRT and VMAT. He has stated that dosimetric methods need peer review, staff awareness, and alertness, which are the necessary checkpoints for reducing the risk of unintended irradiation and a better outcome for the patient [76].

IMRT delivery and VMAT delivery: Previously, 3D conformal plans were the common practice; with the advancement in machines and the introduction of multi-leaf collimators (MLC), IMRT has become the standard mode of delivery. MLC used for intensity modulation and fluence generation come in various forms and are associated with several factors or quality checkpoints. Losasso from the Department of Medical Physics, Memorial Sloan Kettering Cancer Centre, New York, published a report focusing on Varian Millennium 120, mentioning that the factors affecting the dose delivery in clinical fields include mechanical tolerances, motor fatigue, and latency effects, which are also quantified. Alongside that, various other factors like average MLC transmission, interleaf effect, leaf speed, gap position variation, and MLC calibration significantly impact the final dose delivery. They can also become sources of error if not monitored promptly. This leads to poorer clinical outcomes and may lead to increased recurrences [77].

**Figure 1:**
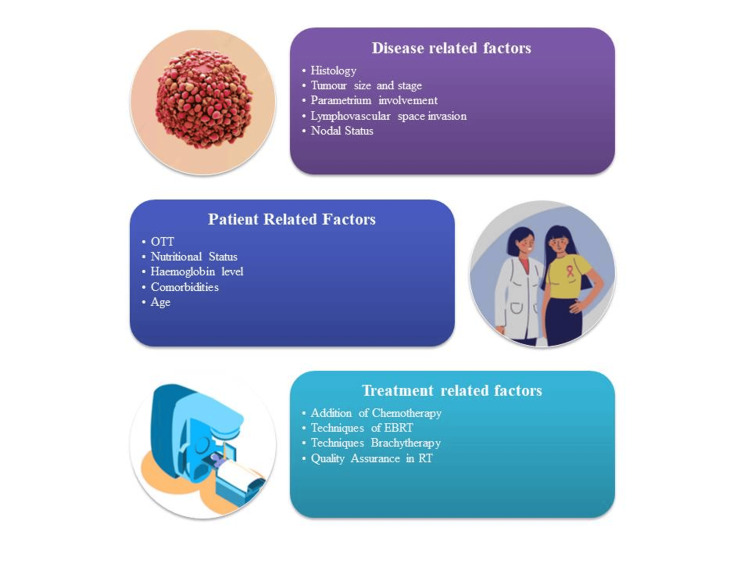
Segregation of various factors Image Credit: Dr. Ashish Uke

**Figure 2:**
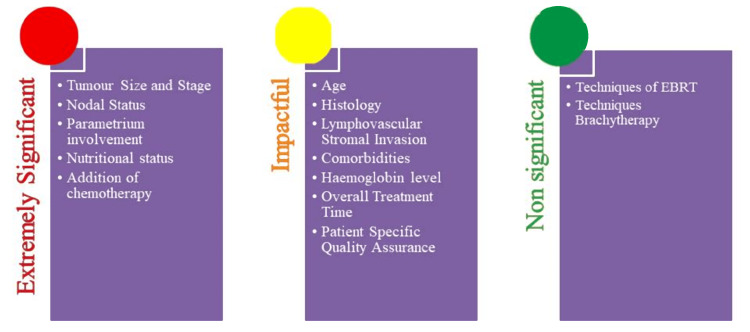
Summary of the factors and their significance Image Credit: Dr. Ashish Uke

Regarding patient-specific quality assurance (PSQA) in IMRT delivery, a multicentric study was performed by Hizam et al. in Malaysia, wherein 40 measurement points were evaluated, of which 18% showed a deviation of more than 5% of the predicted dose. The remaining 82% of the results passed the tolerance level, and most centers passed 95% points of gamma criteria of the 3%/3 mm for planar dose measurement [78]. This indicates that even though the dose calculations and dose delivery are good for most of the plans, a constant check is required to minimize any errors. Table 6 includes studies mapping the dose delivery pattern against the MLC performance. Various factors related to disease, patients, and treatment are summarized in Figure 1 and arranged according to their significance in Figure 2.

**Table 6:** Studies representing the quality assurance results for MLCs w.r.t. dose delivery MLC: multi-leaf collimator; VMAT: volumetric modulated arc therapy; IMRT: intensity-modulated therapy; GYN: gynecological

Sr. no.	Author	Title of the study	Sample	Results
1	Kerns et al. [79]	A multi-institution evaluation of MLC log files and performance in IMRT delivery	85000 Varian MLC treatment logs from six institutions	For the step-and-shoot technique, very small errors were present which increased in dynamic treatment and further increased in VMAT. Restrictive leaf speed can help improve MLC performance
2	Palta et al. [80]	Quality assurance of IMRT	-	IMRT field includes many small irregular off-axis fields and complex beam modulation, which result in higher uncertainties in both planning and delivery processes for which the end user must have a well-defined evaluation criterion for each element of the process
3.	Li et al. [81]	Impact of delivery characteristics on dose delivery accuracy of VMAT on different sites	344 VMAT	Dose delivery accuracy and gamma passing rate are predominantly affected by leaf speed in GYN cancers

## Conclusions

The broad categories of the factors leading to a higher propensity of recurrences post-definitive treatments include disease-related, treatment-related, and patient-related factors, as shown in Figure 1. After reviewing relevant literature and papers, the factors were identified and classified as highly relevant, impactful, and non-significant, as shown in Figure 2. The extremely significant factors include tumor size and stage, nodal involvement, parametrium involvement, nutritional status, and addition of concurrent chemotherapy. Younger age, histology, LVSI, associated comorbidities, hemoglobin level, OTT, and PSQA are significantly impactful factors. Factors that do not have a trend with recurrences are techniques used for EBRT and brachytherapy.
